# Characterization of the complete mitochondrial genome of *Mitjaevia protuberanta*（Hemiptera: Cicadellidae: Typhlocybinae）

**DOI:** 10.1080/23802359.2019.1710601

**Published:** 2020-01-14

**Authors:** Xiaowei Yuan, Can Li, Yuehua Song

**Affiliations:** a School of Karst Science, Guizhou Normal University, State Engineering Technology Institute for Karst Desertification Control, Guizhou, Guiyang, China;; b Guizhou Provincial Key Laboratory for Rare Animal and Economic Insect of the Mountainous Region, Guiyang University, Guizhou, Guiyang, China

**Keywords:** *Mitjaevia protuberanta*, leafhopper, mitochondrial genome

## Abstract

The complete mitogenome of *Mitjaevia protuberanta* (GenBank accession number MN627216) is 14,032 bp (AT: 77.43%) in length, including 13 protein-coding, 22 transfer RNAs, and 2 ribosomal RNAs. All protein-coding genes used ATN as initiation codon except ND5 that used TTG as initiation codon, and TAA, TAG, and T were termination codons. We constructed a phylogenetic tree from 14 species (Hemiptera) based on the nucleotide sequence of 13 mitochondrial protein-coding genes. The phylogenetic analysis results showed that mitochondrial genome of *M. protuberanta* had the same characteristics as other Cicadellidae species.

*Mitjaevia protuberanta* Song et al. (2011) belongs to the genus of *Mitjaevia*, which currently consists of 17 known species around the world (Ghauri 1974; Song et al. 2011). Most of these leafhoppers often have dark spots on the body and are an important agricultural pest, which are widely distributed in the Palearctic and Oriental regions (Korolevskaya 1976). Recently, several investigators established different phylogenetic relationships for Typhlocybinae based on different molecular markers. However, further genomic studies have been hampered by the lack of information on the complete mitogenome of more genera and species form Typhlocybinae. In order to explore its phylogenetic relationship with other leafhoppers, we provide the complete mitochondrial genome of *M. protuberanta* (GenBank: MN627216). Adult specimens of *M. protuberanta* were collected from Fanjing Mountain, Tongren City, Guizhou Province, China (N27°53′, E108°47′). Samples (GZNU-ELS-2019003) have been deposited in the insect specimen room of Guizhou Normal University, Guiyang, China.

The total length of *M. protuberanta* mitochondrial genome is 15,472 bp. The circular mitogenome contains 13 protein-coding genes (PCGs), 2 ribosomal RNA genes (rns and rnl), 22 transfer RNA (tRNA) genes, and a 1416 bp long non-coding AT-rich region. The gene order and orientation of *M. protuberanta* were identical to those observed in other Cicadellidae mitogenomes (Luo et al. 2019; Wang and Xing 2019). The base composition of the genome is as follows: A = 40.01%, T = 37.43%, G = 10.63%, and C = 11.93%, and was biased toward AT (accounting for 88.50%). This mitogenome presented a negative AT-skew (0.033) and a positive GC-skew (–0.058). Twenty-three genes were transcribed on the majority strand (N-strand), whereas the others were oriented on the minority strand (J-strand).

The *M. protuberanta* mitogenome displays gene overlap in 48 bp in 12 locations, the longest 10 bp overlapping exists between *trnS_2_* and *ND1*. Intergenic region has been found at 13 gene junctions, and all of them ranged in the size from 1 to 10 bp. The initial codons for 12 PCGs of *M. protuberanta* were the canonical putative start codons ATN (six with ATG, four with ATA, and two with ATT). Only *ND5* started with TTG. The *COX2* is terminated with a single T residue as the stop codon, *ND5* end with TAT, *CYTB* end with TAG, and remaining 10 PCGs end with TAA. To validate the phylogenetic position of *M. protuberanta*, we constructed the phylogenetic trees of 14 closely related species based on the nucleotide sequences of the 13 core PCGs. The result of phylogenetic tree indicated the close relationship between *M. protuberanta* and *Illinigina* sp. (Figure 1), which is consistent with the phylogenetic analyses of Hemiptera using combined sequence data of mitochondrial and nuclear genes (Wang and Xing 2019).

**Figure 1.**
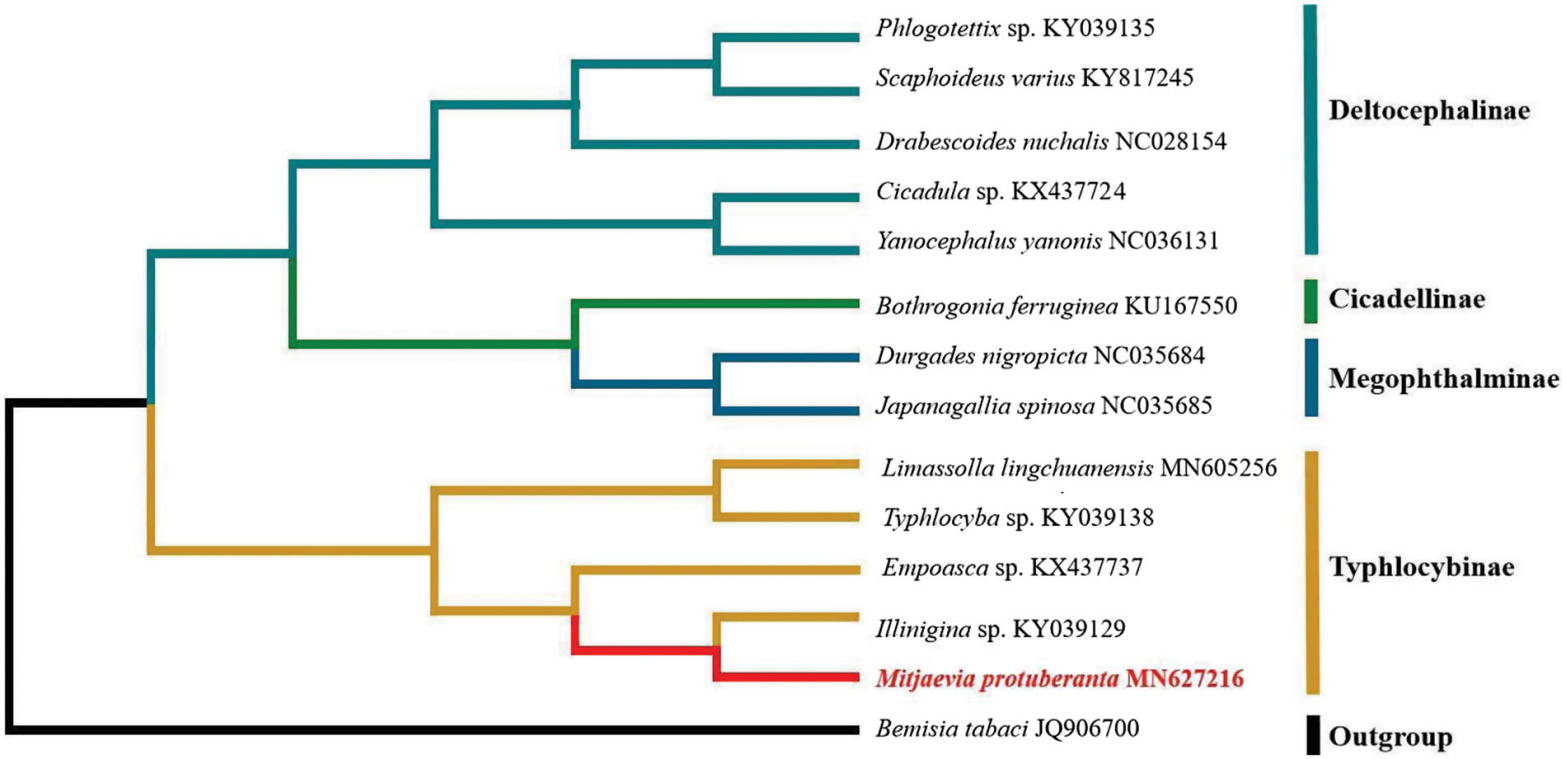
Phylogenetic tree showing the relationship between *Mitjaevia protuberanta* and 13 species (Hemiptera) based on neighbor-joining method.
